# Gut Bacteria Mediate Aggregation Pheromone Release in the Borer Beetle *Trigonorhinus* sp.

**DOI:** 10.3390/insects16100999

**Published:** 2025-09-25

**Authors:** Jinyang Dong, Xiang Yao, Yanru Zhang, Xiuhua Wu, Xinhai Liu, Hongbin Zhang, Haiyan Jiang, Jianli Hou, Jie Yan, Jianing Sun

**Affiliations:** 1 Department of Forest Conservation, Forestry College, Inner Mongolia Agricultural University, Hohhot 010018, China; igl@msn.com (J.D.); tf185865640@126.com (X.Y.); jhydlm@126.com (H.J.); sjn326511@126.com (J.S.); 2 Inner Mongolia Academy of Forestry Sciences, Hohhot 010013, China; wuxiuhua-73@163.com; 3 Ulanqab Institute of Agricultural and Forestry Sciences, Ulanqab 012001, Chinayanjie0705212@126.com (J.Y.); 4 Ulanqab Forestry Protection Station, Ulanqab 012000, China; wlcbzhb@126.com (H.Z.);

**Keywords:** gut microbiome, aggregation pheromone, chemical communication, *Trigonorhinus* sp., host-microbe interaction, *Caragana liouana*, chemical ecology, microbial symbiosis, symbiont-based control

## Abstract

Gut microbial symbionts are increasingly recognized as key modulators of host insect physiology and behavior, yet their role in pheromone-mediated chemical communication remains insufficiently understood. In this study, we investigated the wood-boring beetle *Trigonorhinus* sp., a pest of *Caragana liouana*, to determine the necessity of gut bacteria for male aggregation pheromone release. A combination of antibiotic-mediated bacterial depletion, quantitative PCR, gas chromatography-mass spectrometry (GC-MS), and Y-tube olfactometry was employed. Antibiotic treatment resulted in a marked reduction in gut bacterial load and a concomitant decrease of more than 85% in the emission of two key pheromone components, 2,6,10,14-tetramethylheptadecane and heptacosane. Behavioral assays demonstrated that females no longer exhibited significant attraction to treated males. Furthermore, defined recolonization with a single cultured gut isolate, *Acinetobacter guillouiae*, was sufficient to rescue pheromone emission. This indicates that particular gut taxa, rather than microbial biomass alone, are essential for pheromone biosynthesis. These findings demonstrate a decisive role of gut bacteria in the chemical communication of *Trigonorhinus* sp. and highlight the potential of symbiont-targeted strategies for pest management.

## Simple Summary

An investigation was conducted on the wood-boring beetle *Trigonorhinus* sp., a pest of *Caragana liouana*, to determine the necessity of gut bacteria for male aggregation pheromone release. Using antibiotic depletion, qPCR, GC-MS, and Y-tube olfactometry, it was verified that a marked reduction in gut bacterial load led to more than an 85% decrease in the emission of two key pheromone components (2,6,10,14-tetramethylheptadecane and heptacosane), and females no longer exhibited significant attraction to treated males. Recolonization with a specific gut bacterial isolate, *Acinetobacter guillouiae*, restored pheromone emission to near-control levels, demonstrating a strain-specific effect. These findings demonstrate a decisive role of specific gut taxa in the beetle’s chemical communication and suggest feasible avenues for symbiont-targeted pest management.

## 1. Introduction

The gut microbes of insects can strongly influence their chemical communication. This has become a major research area in entomology. These microbes can control behaviors and bodily functions that scientists once thought were controlled by genes alone [1,2,3,4]. One such insect is the wood-boring weevil *Trigonorhinus* sp. (Anthribidae), a major pest of *Caragana liouana* shrubs in arid regions [5]. This diminutive beetle (2.7–3.5 mm) exhibits a dark-brown to black integument with greyish-white to brown setae and convergent traits with its woody substrate, including distinctly punctate elytra and elongate, multi-segmented antennae that couple camouflage with chemosensory acuity. Sexual dimorphism is subtle but diagnostic: females possess an elongate sixth abdominal sternite, enabling rapid, reliable identification in the field [6].

The damage from *Trigonorhinus* sp. is severe. By killing *C. liouana*, a key plant, it harms the entire dryland ecosystem. Females oviposit on current-year shoots; upon hatching, larvae tunnel into the plant’s veins and create galls. These galls block water flow, which causes the branches to die [7]. These galling processes co-opt host development, redirecting resources toward anomalous tissue growth while provisioning developing larvae. Reproduction peaks from May to August in synchrony with host phenology, suggesting long-term co-adaptation, yet recent range expansions across Ningxia, Inner Mongolia, Shanxi, and Shaanxi indicate that environmental change may be perturbing historical population regulation [8,9,10].

Foundational work integrating morphology, behavior, and chemical ecology has accelerated understanding of this species. Song et al. [7] established taxonomic baselines and damage assessment protocols; Zhang et al. [5] clarified host gall developmental dynamics. Critically, Lei et al. [6] combined Y-tube olfactometry, solid-phase microextraction (SPME), solvent extraction, and gas chromatography–mass spectrometry-electroantennographic detection (GC-MS-EAD) to define an aggregation pheromone system comprised of 2,6,10,14-tetramethylheptadecane and heptacosane. The former is constitutively produced by both mated and unmated males, whereas the latter appears restricted to mated individuals and their frass, implying temporal and physiological regulation of pheromone output.

Studies across insects demonstrate that microbial symbionts can shape pheromone biosynthesis by supplying precursors, enzymatically modifying host compounds, or modulating host gene expression [11,12,13,14,15]. For example, the desert locust *Schistocerca gregaria* relies on *Pantoea agglomerans* for guaiacol and phenol production [16,17], and bark beetles leverage microbial partners to generate complex terpene attractants [18,19]. These advances reframe the evolution of chemical communication and highlight microbiome-informed avenues for pest management [20,21,22]. However, whether and how the gut microbiome governs aggregation pheromone biosynthesis in *Trigonorhinus* sp. has remained untested.

Here, we address this gap by testing the hypothesis that gut bacteria regulate male aggregation pheromone emission in *Trigonorhinus* sp. To test this, we disrupted the beetle’s gut bacteria and then analyzed its pheromones and behavior. Our goal was to see if the microbes directly control pheromone production. This could lead to new pest control strategies that target these microbes.

## 2. Materials and Methods

### 2.1. Insect Collection and Rearing

The study insect was identified as a species of *Trigonorhinus* (Coleoptera: Anthribidae) by the weevil specialist Boris Korotyaev [5]. Further identification to the species level is ongoing.

**Figure 1:**
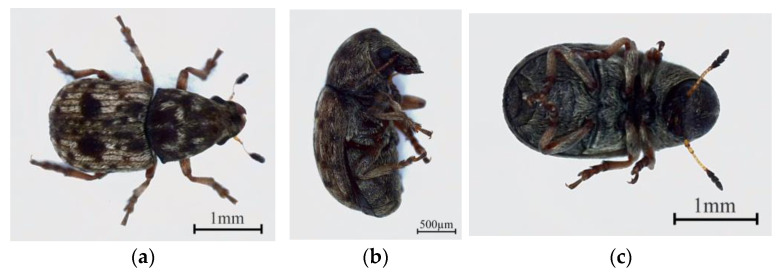
Habitus of adult *Trigonorhinus* sp. (**a**) Dorsal view; (**b**) Lateral view; (**c**) Ventral view.

The insects were acquired by collecting galls from their host plant, *C. liouana*, at a single location in Horinger County, Inner Mongolia (111°51′15″ E, 40°30′48″ N). These galls were transported to the laboratory and held in an incubator at 25 °C ± 2 °C, 70 ± 10% RH, and a 16L:8D photoperiod to facilitate adult emergence. Adults from this stock colony were used for all experiments (Figure 1).

All procedures followed institutional and local guidelines, and no permits were required for this non-protected arthropod.

### 2.2. Gut Dissection and Sample Preparation

Adults were surface-sterilized by immersion in 75% ethanol for 1 min, followed by three rinses in sterile phosphate-buffered saline (PBS; pH 7.2–7.4). Under aseptic conditions on ice, entire guts were dissected using sterile forceps and micro-scissors. A portion of the dissected guts was immediately stored at −80 °C for subsequent DNA extraction and metabolomics analysis, while the remainder was processed fresh for culture-dependent assays. A total of 48 individuals (24 males and 24 females) were used for microbiome analyses.

### 2.3. Antibiotic Treatment Protocol

To generate aposymbiotic insects, ciprofloxacin was incorporated into the sterile artificial diet at a final concentration of 1 mg/mL. Newly emerged males were fed this antibiotic-containing diet for 7 days (antibiotic group, AT). Control males (CK) were fed the same sterile diet without antibiotics. The efficacy of the treatment was verified by qPCR targeting the bacterial 16S rRNA gene and by plating gut homogenates on LB agar (25 °C for 72 h) to confirm the absence of culturable bacteria. A recovery group was established by transferring antibiotic-treated males back to a normal sterile diet for 14 days to assess the restoration of the microbiome and pheromone production. Sample sizes for these experiments were as follows: CK (n = 30), AT (n = 30), and recovery (n = 20).

For microbial reconstitution, isolated bacterial strains were cultured to log phase, washed, and re-suspended in sterile PBS. The suspensions were then evenly incorporated into the sterile artificial diet to create reconstitution groups designated L1–L6, N3, and N4. Beetles were fed these diets for 7 days before analysis. A vehicle-only control, containing sterile PBS mixed into the diet, was also included. The specific strains and consortia corresponding to each label are detailed in the relevant figures and tables.

### 2.4. Culture-Dependent Isolation and Identification of Gut Bacteria

**Figure 2:**
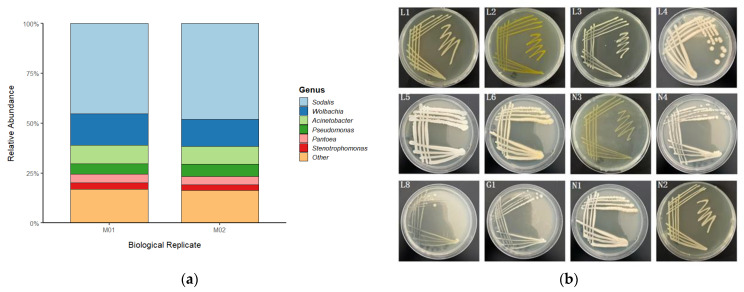
Gut microbiota analysis of *Trigonorhinus* sp. using metagenomic and culture-based methods. (**a**) Relative abundance of the major bacterial genera in the gut of *Trigonorhinus* sp. based on 16S rRNA gene sequencing. (**b**) Colony morphologies of the 12 isolated and purified bacterial strains.

Gut homogenates were plated on Luria-Bertani (LB), Nutrient Agar (NA), and Gao’s No. 1 agar media and incubated at 25 °C. Morphologically distinct colonies were isolated and purified by repeated streaking. Genomic DNA was extracted from each pure isolate, and the 16S rDNA gene was amplified and sequenced. Taxonomic identities were assigned using the BLAST tool (version 2.14.0) against the NCBI database [23]. All isolates were preserved as glycerol stocks at −80 °C. In total, 12 bacterial isolates were obtained (Figure 2b; Table 1), and a neighbor-joining tree was constructed to visualize their phylogenetic relationships (Figure 2b).

### 2.5. 16S rRNA Gene Amplicon Sequencing for Bacterial Community Profiling

16S rRNA gene amplicon sequencing was employed to obtain a broad profile of the bacterial community across multiple individual insects. Total genomic DNA was extracted from individual guts using the FastDNA SPIN Kit (MP Biomedicals, Solon, OH, USA) for Soil [24]. The V3–V4 hypervariable region of the 16S rRNA gene was amplified using the primers 341F/806R [25]. The resulting amplicon libraries were sequenced on an Illumina MiSeq platform (2 × 250 bp paired-end). Extraction blanks and PCR negative controls were included in each sequencing run. Samples with fewer than 10,000 high-quality reads were excluded from further analysis.

### 2.6. Shotgun Metagenomic Sequencing for Unbiased Community Characterization

In a complementary approach, shotgun metagenomic sequencing was used for a deeper, unbiased analysis of the total microbial community. This method allowed for the characterization of all microbial members, including fungi and other non-bacterial taxa, and provided higher-resolution taxonomic and functional data. For this purpose, gut DNA from multiple individuals was pooled to construct two biological replicate libraries (M01 and M02) for shotgun metagenomic sequencing on an Illumina NovaSeq 6000 platform (2 × 150 bp paired-end). After quality control and removal of host-derived reads, the data were used to profile the microbial community composition.

### 2.7. Amplicon and Metagenome Data Processing

Amplicon data were processed using the QIIME 2 pipeline [26]. The DADA2 plugin was used for sequence denoising, merging, and chimera removal to generate amplicon sequence variants (ASVs) [27]. Taxonomy was assigned against the SILVA database [28]. Alpha diversity and beta diversity were calculated. Functional profiles were predicted from the ASV table using PICRUSt2 [29].

Metagenome data were analyzed following quality control and host read removal. Community composition metrics and visualizations, such as Good’s coverage and Principal Component Analysis (PCA), were generated to assess sequencing depth and replicate similarity.

### 2.8. Pheromone Collection and GC-MS Analysis

Aggregation pheromones from individual males (n = 15 per treatment group) were collected via headspace aeration. Each male was enclosed in a glass chamber, and purified air was passed through at a rate of 200 mL/min for 24 h. Volatiles were trapped on a Porapak Q adsorbent (50 mg). The trapped compounds were then eluted with 500 μL of n-hexane containing n-octadecane (10 ng/μL) as an internal standard.

The samples were analyzed on an Agilent 7890B Gas Chromatograph coupled to a 5977B Mass Spectrometer (GC-MS) (Agilent, Santa Clara, CA, USA), equipped with a 5% phenyl methyl siloxane capillary column (30 m × 250 μm × 0.25 μm). An aliquot of 1 μL of each sample was injected in splitless mode with an inlet temperature of 250 °C. High-purity helium (>99.99%) was used as the carrier gas under a constant pressure mode. The oven temperature was programmed as follows: held at 50 °C for 1 min, then ramped at 10 °C/min to 180 °C and held for 2 min, and finally increased at 20 °C/min to 240 °C with a 5 min hold. The total run time was 32 min. The mass spectrometer was operated in electron ionization (EI) mode at 70 eV, with the ion source and quadrupole temperatures maintained at 230 °C and 150 °C, respectively. Mass spectra were acquired in full scan mode over a mass range of 30–600 amu from 5.5 min to 32 min.

Each sample was analyzed with six technical replicates. The amounts of 2,6,10,14-tetramethylheptadecane and heptacosane were quantified relative to the internal standard. To account for variations in body size, pheromone quantities were normalized by the body weight of each male, which was measured to ±0.1 mg prior to collection.

### 2.9. Behavioral Bioassays (Y-Tube Olfactometer)

All behavioral bioassays were conducted in a dedicated testing room under controlled environmental conditions of 24–25 °C and 50–60% relative humidity. To ensure uniform lighting and eliminate external visual cues, the windows were covered with thick, opaque curtains, and illumination was provided by incandescent lamps.

A glass Y-tube olfactometer (stem: 15 cm; arms: 15 cm at a 60° angle) was used for all assays. Purified and humidified air was delivered to each arm at a constant flow rate of 400 mL/min using an atmospheric sampler (Model QC-1S; Beijing Ke’an Labor Protection Technology Co., Ltd., Beijing, China), which integrated both the vacuum pump and flowmeters. For each trial, one arm contained a live male from a specific treatment group as the odor source, while the other arm, serving as a blank control, remained empty.

A single virgin female was gently introduced at the base of the olfactometer’s stem and was observed for a total duration of 5 min. A definitive choice was recorded only if the female moved more than 5 cm into one of the arms and remained there for at least 60 consecutive seconds. Any female that entered an arm but stayed for less than 60 s, or failed to make a definitive choice within the 5 min observation period, was recorded as having “no response”. Each insect was tested only once. For each treatment, 25 responding virgin females were tested.

To prevent positional bias and minimize contamination, several procedures were followed. The sequence of testing different treatment groups was randomized. The positions of the odor source and blank control arms were swapped after every five trials. Furthermore, the entire glass apparatus was thoroughly cleaned with 75% ethanol and oven-dried at 60 °C for 30 min between each individual bioassay to eliminate any residual chemical cues.

### 2.10. Statistical Analysis

**Figure 3:**
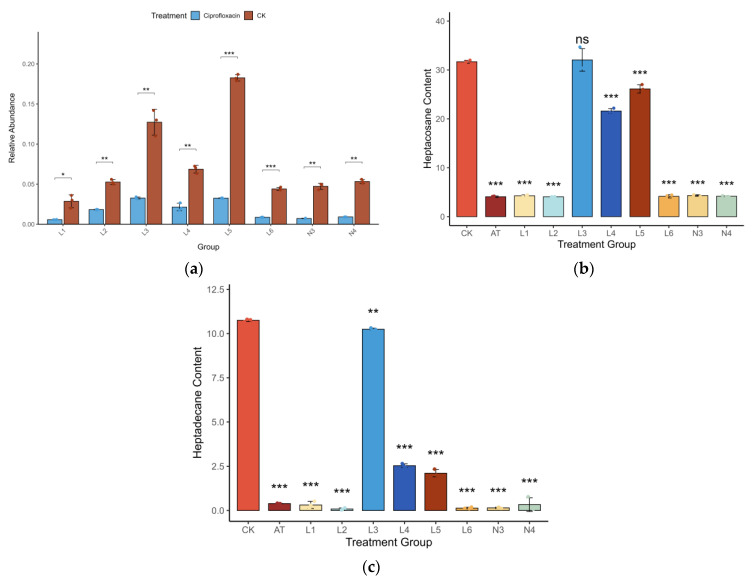
Effects of gut microbiota manipulation on bacterial abundance and aggregation pheromone production in *Trigonorhinus* sp. (CK: control; AT: antibiotic treatment). (**a**) Relative abundance of gut bacteria in different groups, comparing control (CK) and ciprofloxacin-treated conditions. Data are shown as mean ± SD (n = 3). Asterisks indicate significant differences determined by a paired *t*-test (* *p* < 0.05, ** *p* < 0.01, *** *p* < 0.001). (**b**) Heptacosane content (ng/male/24 h) and (**c**) 2,6,10,14-tetramethylheptadecane (heptadecane) content (ng/male/24 h) in each treatment group. Data are shown as mean ± SD (n = 3), with individual data points overlaid. Asterisks denote significant differences compared to the control (CK) group, determined by a one-way ANOVA followed by Dunnett’s test (ns: not significant, *** *p* < 0.001).

All statistical analyses were performed in R (version 4.5.0) [30]. Data normality was assessed using the Shapiro-Wilk test, and appropriate parametric or non-parametric tests were subsequently applied. Differences in alpha diversity were evaluated with the Wilcoxon rank-sum test. Differences in microbiome composition (beta diversity) were tested using PERMANOVA with the ‘adonis2’ function in the ‘vegan’ package [31]. Correlations between microbial taxa and chemical compounds were assessed using Spearman’s rank correlation with false discovery rate (FDR) correction. Y-tube choice data were analyzed using a chi-square test against an expected 50:50 distribution. A p-value of less than 0.05 was considered statistically significant for all tests. Differences in pheromone content among treatment groups (Figure 3b,c) were analyzed using a one-way Analysis of Variance (ANOVA), followed by Dunnett’s post hoc test to compare each treatment group against the control (CK) group. The effect of ciprofloxacin treatment on the relative abundance of each bacterial group (Figure 3a) was assessed using a paired *t*-test.

## 3. Results

### 3.1. Gut Microbiota Composition of Trigonorhinus sp.

To comprehensively characterize the gut microbiota of *Trigonorhinus* sp., we employed a combination of metagenomic and traditional culture-based techniques.

The metagenomic analysis revealed a community structure dominated by a few major genera (Figure 2a). Across all samples, *Sodalis* was the absolute dominant genus, with a relative abundance approaching 50%, followed by *Wolbachia* with a relative abundance of approximately 15%. Additionally, genera such as *Acinetobacter*, *Pseudomonas*, *Pantoea*, and *Stenotrophomonas* also constituted a certain proportion of the community. The microbial composition of the two biological replicates demonstrated high consistency, indicating a relatively stable gut community structure.

In a parallel, culture-dependent approach, gut homogenates were plated on various agar media. A total of 12 bacterial strains with distinct colony morphologies were isolated and purified (Figure 2b). Through 16S rDNA gene sequencing and BLAST analysis for definitive identification, these 12 isolates were identified as eight distinct species: *Acinetobacter guillouiae* (3 strains), *Stenotrophomonas lactitubi*, *Pantoea plantarum*, *Mammaliicoccus sciuri* (3 strains), *Pseudomonas allii*, *Comamonas sediminis*, *Pantoea endophytica*, and *Brucella pseudogrignonensis*.

Although the two methods differ in resolution, their results show important consistency and complementarity. Several genera obtained through culturing, such as *Acinetobacter*, *Stenotrophomonas*, *Pantoea*, and *Pseudomonas*, were also detected in the metagenomic results (Figure 2a), confirming their presence as members of this gut ecosystem. The sequencing technology revealed the presence of difficult-to-culture dominant symbionts like *Sodalis* and *Wolbachia*, while the culture-based method successfully yielded viable strains for subsequent functional studies.

### 3.2. Gut Bacteria Are Essential for Pheromone Release

To investigate the relationship between gut microbiota and pheromone production in *Trigonorhinus* sp., we quantified both bacterial abundance and pheromone levels under different treatments. qPCR analysis showed that ciprofloxacin treatment led to a dramatic reduction in gut bacterial abundance across all tested groups, with decreases ranging from 65% to over 90% compared to the control (Figure 3a). This confirmed the effective elimination of gut microbiota following antibiotic exposure.

Subsequent GC-MS analyses revealed that the elimination of gut bacteria resulted in a marked suppression of aggregation pheromone release (Figure 3b,c). In the antibiotic-treated (AT) group, both heptacosane and heptadecane levels were drastically reduced (averaging 4.07 ng and 0.39 ng, respectively), representing a reduction of over 85% compared to the control (CK) group, which showed the highest pheromone levels (averaging 31.68 ng and 10.75 ng, respectively). Notably, only the L3 group, which was recolonized with a specific microbial community, exhibited a full recovery of pheromone production, with levels comparable to the control. The L4 and L5 groups showed partial recovery, while other groups (L1, L2, L6, N3, N4) failed to restore pheromone synthesis, with levels remaining as low as those in the AT group. These results indicate that gut microbiota are indispensable for pheromone biosynthesis in male beetles, with significant differences observed among the treatment groups for both heptacosane (ANOVA, F(9, 20) = 718.2, *p* < 0.001) and heptadecane (ANOVA, F(9, 20) = 2047, *p* < 0.001). Furthermore, they suggest that only certain core bacterial taxa are capable of fully restoring this function.

### 3.3. Characterization of the Gut Microbiome in Trigonorhinus sp.

We studied the gut bacteria of a beetle called *Trigonorhinus* sp. We used two separate samples, named M01 and M02. Our sequencing was very thorough, capturing over 99.99% of the bacteria in both samples.

The number of different species in the gut was high. Both samples had similar diversity, with a Shannon index around 4.2. However, the number of unique species was different. We found 2075 species in M01 and 2288 in M02. This shows that even though the samples came from the same group, there was some natural variety.

**Figure 4:**
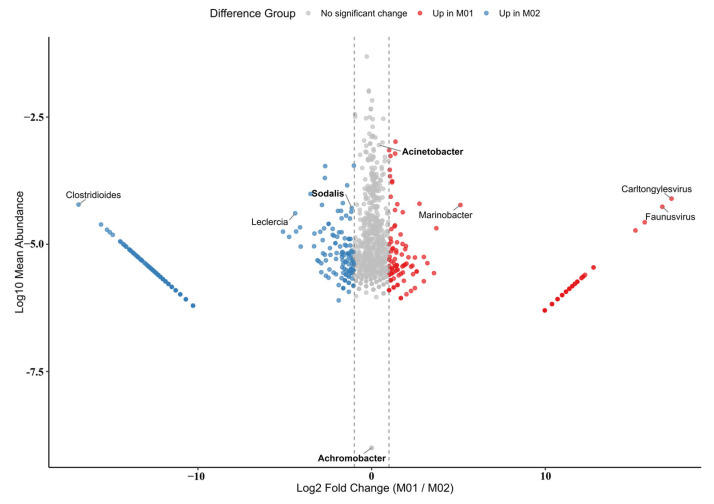
Differential Abundance Analysis of Microbial Genera Between *Trigonorhinus* sp. The X-axis shows the Log2 Fold Change, measuring how much more abundant a genus is in one sample versus the other. Points far to the left are much more abundant in M02, while points far to the right are much more abundant in M01. The Y-axis represents the Log10 Mean Abundance, so genera with a higher overall abundance appear at the top of the plot. The colors on the plot highlight the direction of change: blue points are enriched in M01, red points are enriched in M02, and gray points show no significant change. Additionally, the label text indicates importance: bolded labels are used to highlight the genera with the least significant changes, while unbolded labels point to the genera with the most significant changes.

A visual analysis called PCA showed a clear difference between the two samples (Figure 4). M01 was on one side of the plot, and M02 was on the other. This difference was statistically significant. The plot shows that many bacteria were much more common in one sample than the other. For example, some bacteria like *Clostridioides* were much more abundant in M01. Other bacteria, like *Faunusvirus* and *Carltongylesvirus*, were more abundant in M02.

Despite these differences, the main types of bacteria were the same in both samples. The most common bacteria belonged to four main groups: Proteobacteria, Firmicutes, Bacteroidetes, and Actinobacteria. This is a common pattern in insect guts.

At a more detailed level, we found that a few genera dominated the community. Sodalis was the most common genus overall. Other important genera, like *Acinetobacter*, *Leclercia*, and *Achromobacter*, were also present. The consistent presence of these bacteria suggests they are a stable and important part of the beetle’s gut.

### 3.4. Behavioral Consequences of Microbiome Depletion

In Y-tube olfactometer assays, females showed a strong and significant preference for the odor of control (CG) males over a blank control (χ^2^ = 31.183, *p* < 0.001). This attraction was completely abolished when aposymbiotic (AT) males were used as the odor source, with females showing no preference between the odor of the AT male and the blank control (χ^2^ = 0.065, *p* = 0.799) (Table 2).

## 4. Discussion

This study reveals that the gut microbial community is indispensable for the production of aggregation pheromones in the borer beetle Trigonorhinus sp. Our integrated approach, combining microbial depletion and targeted recolonization with chemical and behavioral analyses, establishes a clear causal link between gut bacteria and the host’s chemical communication system. These findings reveal a multi-layered symbiosis in which gut bacteria provide nutritional support and govern host reproduction. This key function of pheromone synthesis is highly specific to *Acinetobacter guillouiae*, while other symbionts like *Mammaliicoccus sciuri* and *Pseudomonas allii* make partial contributions, demonstrating functional redundancy.

Our characterization of the *Trigonorhinus* sp. gut microbiome revealed a community dominated by the phylum Proteobacteria, which is consistent with findings in other wood-boring beetles that subsist on recalcitrant plant diets [32,33,34,35]. Notably, there was a discrepancy between our culture-dependent and metagenomic sequencing results [36,37,38,39,40,41]. While metagenomic analysis identified *Sodalis* as the most abundant genus, our culture-based approach successfully isolated twelve distinct strains, including members of *Acinetobacter*, *Pantoea*, and *Pseudomonas*. This highlights a common challenge in microbiology, where the most abundant taxa may not be the most easily culturable or, critically, the most functionally relevant for a specific metabolic task.

The central finding of our study is the definitive link between the gut microbiome and pheromone production. The near-complete cessation of pheromone release and the corresponding loss of behavioral attraction in aposymbiotic beetles provide unequivocal evidence for this dependency. Most significantly, the recolonization experiments demonstrated a high degree of functional specificity. The reintroduction of a single bacterial isolate, *Acinetobacter guillouiae* (L3), was sufficient to fully restore pheromone emission to control levels. In contrast, other isolates resulted in only partial (L4, L5) or no recovery at all. This strongly indicates that pheromone biosynthesis is not a generic microbial function but is dependent on specific taxa possessing the necessary metabolic capabilities.

The complete rescue by *A. guillouiae* points to this species as a key player in the metabolic partnership. Members of the genus *Acinetobacter* are renowned for their versatile metabolic capabilities, particularly in the degradation and synthesis of various hydrocarbons and lipids [42,43,44,45,46,47]. Given that the aggregation pheromones of *Trigonorhinus* sp. are long-chain alkanes (heptacosane and a tetramethylheptadecane), it is plausible that *A. guillouiae* contributes essential enzymes or precursors for the biosynthesis or modification of these compounds, a hypothesis that warrants direct investigation in future studies. The partial recovery induced by *Mammaliicoccus sciuri* (L4) and *Pseudomonas allii* (L5) suggests these bacteria may also possess some, albeit less efficient, relevant metabolic pathways.

From an applied perspective, the obligate dependence of *Trigonorhinus* sp. on specific gut symbionts for chemical communication presents a novel and promising target for pest management. Strategies aimed at disrupting this crucial symbiotic relationship, a concept known as “symbiont-based control,” could offer a highly specific and environmentally benign alternative to conventional insecticides [3,48,49,50,51]. This could involve the development of molecules that specifically inhibit key microbial pathways or the use of para-transgenesis to introduce engineered bacteria that interfere with pheromone production, thereby disrupting mating and controlling pest populations [52,53,54].

## 5. Conclusions

In conclusion, our study reveals that the gut bacterial community plays an indispensable role in the aggregation pheromone biosynthesis of the borer beetle *Trigonorhinus* sp. We demonstrate a clear functional link between the microbiome and the host’s chemical communication system. Specifically, we identified a single bacterial species, *Acinetobacter guillouiae*, capable of fully restoring pheromone production in symbiont-depleted beetles, highlighting a sophisticated and highly specific host-microbe co-adaptation. These findings not only fundamentally advance our understanding of the evolution of chemical communication and insect-microbe symbiosis but also open up new avenues for the development of innovative and sustainable symbiont-targeted strategies for the control of this important pest.

## Figures and Tables

**Table 1:** 16S rDNA sequence alignment of intestinal bacteria in *Trigonorhinus* sp.

Strain No.	Most Closely Related Strain	Similarity (%)	Accession Number
L1	*Stenotrophomonas lactitubi*	99.79	NR179509
L2	*Pantoea plantarum*	99.79	NR104943
L3	*Acinetobacter guillouiae*	99.50	NR117626
L4	*Mammaliicoccus sciuri*	100	NR025520
L5	*Pseudomonas allii*	100	NR179337
L6	*Comamonas sediminis*	99.14	NR149789
L8	*Mammaliicoccus sciuri*	100	NR025520
N1	*Mammaliicoccus sciuri*	100	NR025520
N2	*Acinetobacter guillouiae*	99.5	NR117626
N3	*Pantoea endophytica*	99.71	NR178843
N4	*Brucella pseudogrignonensis*	100	NR042589
G1	*Acinetobacter guillouiae*	99.5	NR117626

**Table 2:** Detailed results of Y-tube olfactometer bioassays showing the olfactory responses of *Trigonorhinus* sp. males and females to odor sources from different treatment groups.

**Treatment Group**	**Responding Insect**	**Chose Odor Source**	**Chose Control Source**	**No Response**	**Response Rate (%)**	**Chi-Square (χ^2^)**	***p*-Value**
CG	Female	63	14	13	81.82	31.183	<0.001
	Male	66	15	9	81.48	32.111	<0.001
AT	Female	30	32	28	48.39	0.065	0.799
	Male	32	30	28	51.61	0.065	0.799
L1	Female	26	28	36	48.15	0.074	0.785
	Male	27	30	33	47.37	0.158	0.691
L2	Female	24	22	44	52.17	0.087	0.768
	Male	23	22	45	51.11	0.022	0.881
L3	Female	64	16	10	80	28.8	<0.001
	Male	66	15	9	81.48	32.11	<0.001
L4	Female	48	20	22	70.59	11.529	0.001
	Male	49	16	25	75.38	16.754	<0.001
L5	Female	58	18	14	76.32	21.053	<0.001
	Male	61	20	9	75.31	20.753	<0.001
L6	Female	29	26	35	52.73	0.164	0.686
	Male	30	28	32	51.72	0.069	0.793
N3	Female	31	30	29	50.82	0.016	0.898
	Male	32	30	28	51.61	0.065	0.799
N4	Female	27	25	38	51.92	0.077	0.782
	Male	28	25	37	52.83	0.17	0.68

## Data Availability

The sequencing data presented in this study are openly available in Figshare at https://doi.org/10.6084/m9.figshare.30196861.
